# High neutrophil/lymphocyte ratio at cancer diagnosis predicts incidence of stroke in cancer patients

**DOI:** 10.1093/braincomms/fcab071

**Published:** 2021-04-09

**Authors:** Tomohiro Kawano, Tsutomu Sasaki, Yasufumi Gon, Takaya Kitano, Hideaki Kanki, Kenichi Todo, Munehisa Shimamura, Yasushi Matsumura, Ao Huang, Satoshi Hattori, Hideki Mochizuki

**Affiliations:** 1 Department of Neurology, Osaka University Graduate School of Medicine, Suita, Osaka, 565-0871, Japan; 2 Department of Health Development and Medicine, Osaka University Graduate School of Medicine, Suita, Osaka, 565-0871, Japan; 3 Department of Medical Informatics Science, Osaka University Graduate School of Medicine, Suita, Osaka, 565-0871, Japan; 4 Department of Biomedical Statistics, Osaka University Graduate School of Medicine, Suita, Osaka, 565-0871, Japan; 5 Open and Transdisciplinary Research Initiatives, Osaka University, Suita, Osaka, 565-0871, Japan

**Keywords:** cancer-associated stroke, neutrophil-to-lymphocyte ratio, ischaemic stroke

## Abstract

Although cancer increases the incidence and severity of ischaemic stroke, there is no reliable method for predicting ischaemic stroke in cancer patients. To evaluate the prognostic capacity of the neutrophil-to-lymphocyte ratio at cancer diagnosis for predicting the incidence of ischaemic stroke, we used a hospital-based cancer registry that contained clinical data from all patients treated for cancer at Osaka University Hospital between 2007 and 2015. The neutrophil-to-lymphocyte ratio was calculated after dividing absolute neutrophil counts by absolute lymphocyte counts. These counts were obtained within 1 month after cancer diagnosis. The primary endpoint was new-onset ischaemic stroke within 2 years after cancer diagnosis. Of the 18 217 included cancer patients (median age: 65.2 years), 69 (0.38%) had ischaemic stroke. Unadjusted Cox regression analysis stratified by cancer site demonstrated that each 1-unit increase in the neutrophil-to-lymphocyte ratio was associated with a significant 7.2% increase in the risk of an ischaemic stroke event (95% confidence interval 1.041–1.103, *P* < 0.001). Survival tree analysis and the Kaplan–Meier method suggested that patients with and without atrial fibrillation who had increased neutrophil-to-lymphocyte ratios had a higher risk of ischaemic stroke. Multivariate Cox proportional hazard models, adjusted for cancer site and stage, revealed that patients with high neutrophil-to-lymphocyte ratios (>15) had higher ischaemic stroke risk than patients with low neutrophil-to-lymphocyte ratios (<5). This was true among cancer patients both with (hazard ratio 11.598; 95% confidence interval 0.953–141.181) and without (hazard ratio 7.877; 95% confidence interval 2.351–26.389) atrial fibrillation. The neutrophil-to-lymphocyte ratio at cancer diagnosis is associated with the incidence of ischaemic stroke among cancer patients and might thus be useful for identifying patients at high risk of ischaemic stroke, allowing us to guide future preventive interventions.

## Teaser

Kawano et al. report that the elevated neutrophil-to-lymphocyte ratio at cancer diagnosis is associated with the incidence of ischaemic stroke within 2 years after cancer diagnosis, suggesting that neutrophil-to-lymphocyte ratio at cancer diagnosis might be useful for identifying patients at high risk of ischaemic stroke.

## Graphical Abstract

**Graphical Abstract:**
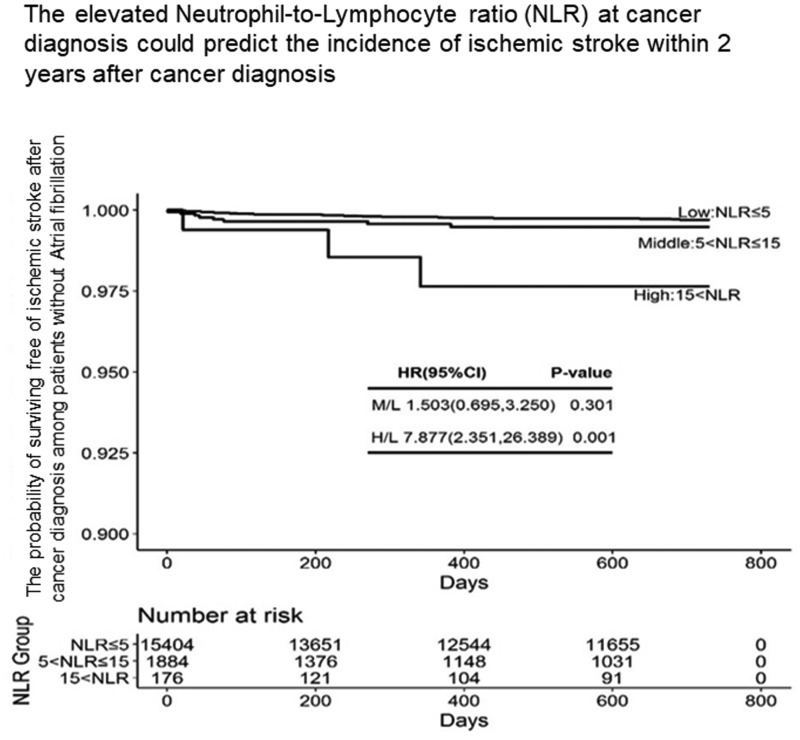

## Introduction

The risk of stroke among cancer patients is more than twice that of the general population, and rises with longer follow-up time.^1^ Moreover, patients with cancer have more severe neurological deficits and greater mortality following stroke than patients without cancer.^2^^,^^3^ The clinical features of stroke patients with cancer (cancer-associated stroke) are multiple vascular lesions, cryptogenic stroke, and increased D-dimer and C-reactive protein (CRP) levels.^4^ One probable contributor to cancer-associated stroke is cancer-associated coagulopathy.^5^ However, the exact pathophysiology of ischaemic stroke in cancer patients remains unclear, as do appropriate preventive strategies. An easy-to-measure and reliable marker is therefore needed to identify patients with a high risk of future stroke.

In recent years, the neutrophil-to-lymphocyte ratio (NLR) has been reported to reflect cancer-related inflammatory responses and have prognostic value in patients with various cancer types.^6^^,^^7^ The NLR is easily calculated by dividing the number of neutrophils by the number of lymphocytes, measured from peripheral blood samples. A high NLR is associated with poor prognosis in patients with large-vessel occlusion stroke or acute intracranial haemorrhage.^8^^,^^9^ Thus, the NLR might also reflect increased inflammatory responses that are evoked by ischaemic stroke and intracerebral haemorrhage. We, therefore, hypothesized that high NLR might be associated with ischaemic stroke in cancer patients, because ischaemic stroke and cancer share some underlying mechanisms, including hypoxia and inflammation.^10^^,^^11^ In this study, we examined whether NLR at cancer diagnosis can predict ischaemic stroke in cancer patients.

## Materials and methods

This study complied with the Declaration of Helsinki for investigations involving humans, and was approved by the institutional review board of Osaka University Hospital.

### Study population

We used patient data from the Osaka University cancer registry. The Osaka University cancer registry is a hospital-based cancer registry that includes patients with cancer diagnosed at the hospital, as well as patients with cancer who came to the hospital after being diagnosed elsewhere. From 2007 to 2015, 27 932 patients were enrolled in this registry. A validated algorithm was used to identify ischaemic stroke events within 2 years of cancer diagnosis; the detailed procedures have been described elsewhere.^12^^,^^13^ We excluded patients who were not followed up at Osaka University Hospital, had a brain tumour, did not have cancer stage records or did not undergo blood examination within 1 month after their cancer diagnosis. A total of 18 217 patients were included in the final analysis.

### NLR assessment

The NLR was calculated by dividing the number of neutrophils by the number of lymphocytes. The neutrophil and lymphocyte counts came from blood samples that were obtained within 1 month after cancer diagnosis or of registration in the Osaka University cancer registry.

### Statistical analysis

The primary aim of this research was to assess and characterize the capacity of the NLR at diagnosis to predict the risk of ischaemic stroke within 2 years from cancer diagnosis. We, therefore, analysed the time to stroke, which was defined as the time period from the date of inclusion in the study until the date a stroke event occurred. The date of inclusion was defined as the date of cancer diagnosis, or the date of the patient’s first visit to Osaka University Hospital (for patients referred after diagnosis). The stroke date was identified according to the algorithm described in the study population section. Follow-up was stopped at the time of death, the last visit to our hospital or 2 years after the cancer diagnosis.

Patient characteristics at enrolment were summarized using medians and interquartile ranges for continuous variables, and as frequencies and percentages for categorical variables.

For an overall understanding of the prognosis capacity of each factor, univariate analyses were conducted using the Cox proportional hazards model. The survival tree technique was also applied, which gives insightful pictures of prognosis in a very simple way by generating recursively subgroups of homogeneous prognosis.^14–16^ At first, the entire population was divided into two groups of the most distinct prognosis capacities with the two-group log-rank test searched over all variables [sex, hypertension, dyslipidaemia, diabetes mellitus, atrial fibrillation (Af), smoking, drinking, NLR, age, white blood cells (WBCs), neutrophils, lymphocytes, CRP, albumin and haemoglobin (Hb)] and all possible cut-off values. Each of the resulting subgroups were recursively partitioned into further subgroups of the factors that most markedly separated subjects’ prognosis using the log-rank test. This process was continued until some statistical criterion met to avoid too precise partitioning and then too unstable estimates due to small number of subjects in some subgroups. To describe and characterize prognosis capacities of subgroups identified by the survival tree analysis, the Kaplan–Meier method was also applied. We also applied the Cox proportional hazards model with spline-based non-linear covariate effects of NLR. The risk of ischaemic stroke may also be dependent on cancer site and cancer stage, meaning that these may be confounding factors for the association between NLR and ischaemic stroke. We, therefore, conducted Cox regression analysis adjusting for cancer site and stage, as well as prognosis factors for ischaemic stroke. Our data contained a wide range of cancer sites, and a very small number of ischaemic stroke events were observed per cancer site (Supplementary Table 1). Furthermore, the meaning of stage was not consistent among cancer sites. Thus, rather than including these factors as explanatory variables in the Cox regression, we stratified the baseline hazard function according to cancer type, defined by a combination of cancer site and cancer stage (advanced for stages III or IV, and non-advanced for stages 0–II). This stratification is meaningful despite the inconsistent definitions of stage among cancer sites; and has been previously used by Kitano et al.^12^ Prognosis factors for ischaemic stroke were also adjusted as potential confounding factors by including as explanatory variables in the Cox regression; diabetes mellitus, hypertension, dyslipidaemia and drinking history. We conducted these analyses separately by Af status. Propensity-score based confounder adjusted analysis was also conducted to address robustness of the confounder-adjustment by the Cox regression, and a subgroup analysis was conducted as sensitivity analysis.

Associations between stage and NLR were examined by calculating the geometric means and confidence intervals of each cancer stage (advanced/non-advanced) by cancer site. We applied two-way analysis of variance (ANOVA) for the cancer site and stage to the log-transformed NLR, to account for the skewed distribution of NLR.

Associations of NLR with CRP, body mass index (BMI), and D-dimer were also examined by Spearman rank correlation.

Statistical analyses were performed using JMP14.3.0 software (SAS Institute, Inc., Cary, NC, USA) and R version 3.6.0 (R core team). R packages of *Party* and *Survival* were used for the survival tree analysis. In this study, *P*-value <0.05 was considered statistically significant.

### Data availability

The data that support the findings of this study are available from the corresponding author upon request.

## Results

### Study population

Of the 18 217 cancer patients enrolled (median age: 65.2 years), 69 (0.38%) had ischaemic stroke. Supplementary Table 2 shows the breakdown of cancer sites among patients with ischaemic stroke. These 69 ischaemic stroke events were characterized as follows. The median (interquartile range) time from cancer diagnosis to ischaemic stroke was 187 (48.5–382) days. As shown in Supplementary Fig. 1A, the distribution peaked in 100 days after cancer diagnosis. Next, we examined the frequency of ischaemic stroke adjusted by the number of cancer survivors. In accordance with the previous report,^17^ the event rate of ischaemic stroke remained nested around the time of cancer diagnosis (Supplementary Fig. 1B). Supplementary Tables 1 and 2 summarize the ischaemic stroke incidence among cancer patients, and patients’ clinical characteristics are shown in Table 1.

**Table 1:** Clinical characteristics of the cancer patients

	Af group (*n *=* *753)	Non-Af group (*n *=* *17 464)	Total (*n *=* *18 217)
Age, years	72 [66–78]	65 [54–73]	65 [55–73]
Male (%)	543 (72)	8805 (50)	9348 (51)
Stage of cancer			
* *Non-advanced (%) (0, I, and II)	500 (69)	11548 (69)	12048 (69)
Advanced (%) (III and IV)	222 (31)	5136 (31)	5358 (31)
Hypertension (%)	416 (55)	3953 (23)	4369 (24)
Dyslipidaemia (%)	239 (32)	2517 (14)	2756 (15)
Diabetes mellitus (%)	315 (42)	3422 (20)	3737 (21)
Smoking (%)	220 (37)	3791 (32)	4011 (33)
Drinking history (%)	130 (17)	1315 (8)	1445 (8)
BMI	22.9 [20.4–24.9]	22.2 [20.0–24.4]	22.2 [20.0–24.5]
WBCs, ×10^3^/µl	6.2 [5.0–7.7]	6.2 [5.0–7.7]	6.2 [5.0–7.7]
Neutrophils, ×10^3^/µl	3.9 [3.0–5.2]	3.9 [2.9–5.1]	3.9 [2.9–5.1]
Lymphocytes, ×10^3^/µl	1.5 [1.2–2.0]	1.6 [1.2–2.0]	1.6 [1.2–2.0]
NLR	2.5 [1.8–3.7]	2.4 [1.7–3.5]	2.4 [1.7–3.5]
CRP, mg/dl	0.23 [0.08–0.91]	0.18 [0.07–0.84]	0.18 [0.07–0.84]
Alb, mg/dl	3.9 [3.6–4.2]	4 [3.6–4.3]	4 [3.6–4.3]
Hb, g/dl	13.2 [11.7–14.4]	13.2 [12–14.2]	13.2 [12–14.2]

Data are given as median [interquartile range] or number (percentage).

Af, atrial fibrillation; Alb, albumin; BMI, body mass index; CRP, C-reactive protein; Hb, haemoglobin; NLR, neutrophil-to-lymphocyte ratio; WBC, white blood cell.

### Higher NLRs were associated with higher risk of ischaemic stroke

Table 2 summarizes the univariate Cox regression analyses stratified by cancer types for NLR, as well as other baseline characteristics and clinical variables. NLR was significantly associated with ischaemic stroke (*P* < 0.001), indicating that patients with higher NLR had greater risk of ischaemic stroke. Af was also significantly associated with ischaemic stroke (*P* < 0.001). Older age, hypertension, dyslipidaemia, diabetes mellitus and neutrophil counts were all positively associated with increased risk of ischaemic stroke in cancer patients. In contrast, lymphocyte counts and Hb levels were negatively associated with ischaemic stroke risk.

**Table 2:** Univariate Cox regression analyses stratified by cancer site

Variables	HR (95% CI)	*P-*value
NLR, per 1 increase	1.072 (1.041–1.103)	<0.001
Age, years, per 1 increase	1.052 (1.027–1.078)	<0.001
Male	1.099 (0.625–1.933)	0.744
Atrial fibrillation	6.025 (3.407–10.656)	<0.001
Hypertension	3.125 (1.935–5.047)	<0.001
Dyslipidaemia	2.927 (1.777–4.820)	<0.001
Diabetes mellitus	2.689 (1.649–4.385)	<0.001
Smoking	0.689 (0.386–1.230)	0.208
Drinking history	1.124 (0.512–2.470)	0.771
BMI (kg/m^2^)	1.004 (0.932–1.082)	0.909
WBCs, ×10^3^/µl, per 1 increase	1.004 (0.989–1.019)	0.619
Neutrophils, ×10^3^/µl, per 1 increase	1.032 (1.008–1.055)	0.007
Lymphocytes, ×10^3^/µl, per 1 increase	0.967 (0.941–0.993)	0.013
CRP, mg/dl, per 1 increase	1.052 (0.964–1.149)	0.255
Alb, mg/dl, per 1 increase	0.906 (0.713–1.151)	0.417
Hb, g/dl, per 1 increase	0.826 (0.728–0.937)	0.003

Alb, albumin; BMI, body mass index; CI, confidence interval; CRP, C-reactive protein; Hb, haemoglobin; HR, hazard ratio; NLR, neutrophil-to-lymphocyte ratio; WBC, white blood cell.

### Higher NLRs were correlated with increased CRP and D-dimer levels

We examined the association of NLR with CRP and D-dimer levels among cancer patients because CRP and D-dimer have been reported to have relationship with cancer-associated stroke.^4^^,^^5^Supplementary Fig. 2 shows there was a significant positive correlation of NLR with CRP (*ρ* = 0.3190, *P<* 0.0001) and D-dimer levels (*ρ* = 0.2315, *P<* 0.001).

### NLR was negatively associated with BMI

Next, we examined the correlation between NLR and BMI among cancer patients because we previously reported that malnutrition due to cancer was associated with the development of cancer-associated stroke.^18^Supplementary Fig. 3 shows there was a significant negative correlation of NLR with BMI (*ρ* = −0.1181, *P<* 0.0001).

### Af and NLR were important variables for ischaemic stroke risk

**Figure 1:**
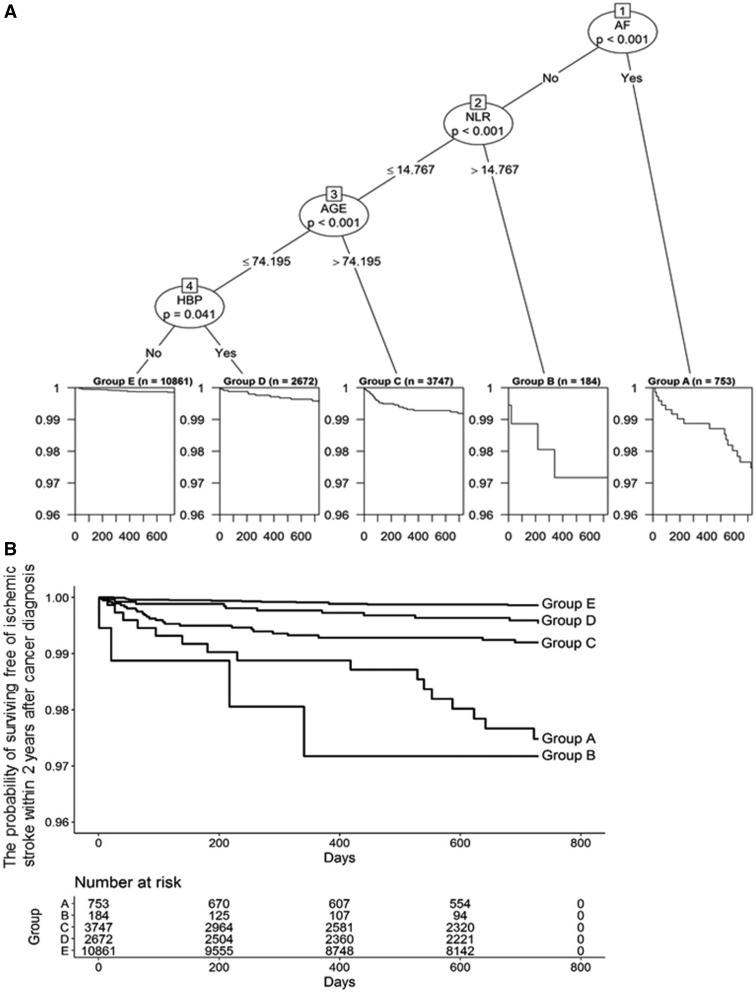
**Patients with Af and with high NLR had higher cumulative rates of ischaemic stroke than other subgroups**. Five prognosis groups identified by the survival tree technique with the log-rank tests (**A**) and Kaplan–Meier plots (**B**). AF, atrial fibrillation; NLR, neutrophil-to-lymphocyte ratio; HBP, hypertension.

We applied the survival tree analysis. Figure 1A shows the tree constructed from the candidate variables of age, Af, hypertension, dyslipidaemia, diabetes mellitus and NLR. The study population was divided into five subgroups of different prognosis capacities (Fig. 1A). Af was selected as the first variable, to discriminate the patients into two subgroups. Thus, the presence of Af was the most important factor for determining heterogeneity in patient prognosis. This result is consistent with current knowledge; Af is the most common cardiac arrhythmia among cancer patients and a major cause of cardioembolic stroke.^19^^,^^20^

No further prognostic factors were suggested for cancer patients with Af (Fig. 1A). However, for patients without Af, NLR, age and hypertension seemed useful for prognosis discrimination. We thus analysed the association between these variables and ischaemic stroke risk in both Af (Af group) and non-Af (non-Af group) patients. Figure 1A indicates that a cut-off value of 14.767 might help to discriminate the prognosis of non-Af patients. An age cut-off value of 74.195 years and the presence of hypertension were also suggested to be useful for discriminating prognosis in non-Af patients. We also examined the incidence of ischaemic stroke and clinical characteristics in the Af and non-Af groups (Table 1 and Supplementary Table 1).

Figure 1B shows the Kaplan–Meier estimates for the survival curves of the five subgroups identified in the survival tree analysis. Patients with Af [Group A (*n *=* *753)] and with high NLR [Group B (*n *=* *184)] had higher cumulative rates of ischaemic stroke than the other subgroups (Fig. 1B). This indicates that Af and high NLR are important variables for the risk of ischaemic stroke. Of these variables, patients with Af had the most independent risk.

### High NLR predicted ischaemic stroke in cancer patients, especially those without Af

**Figure 2:**
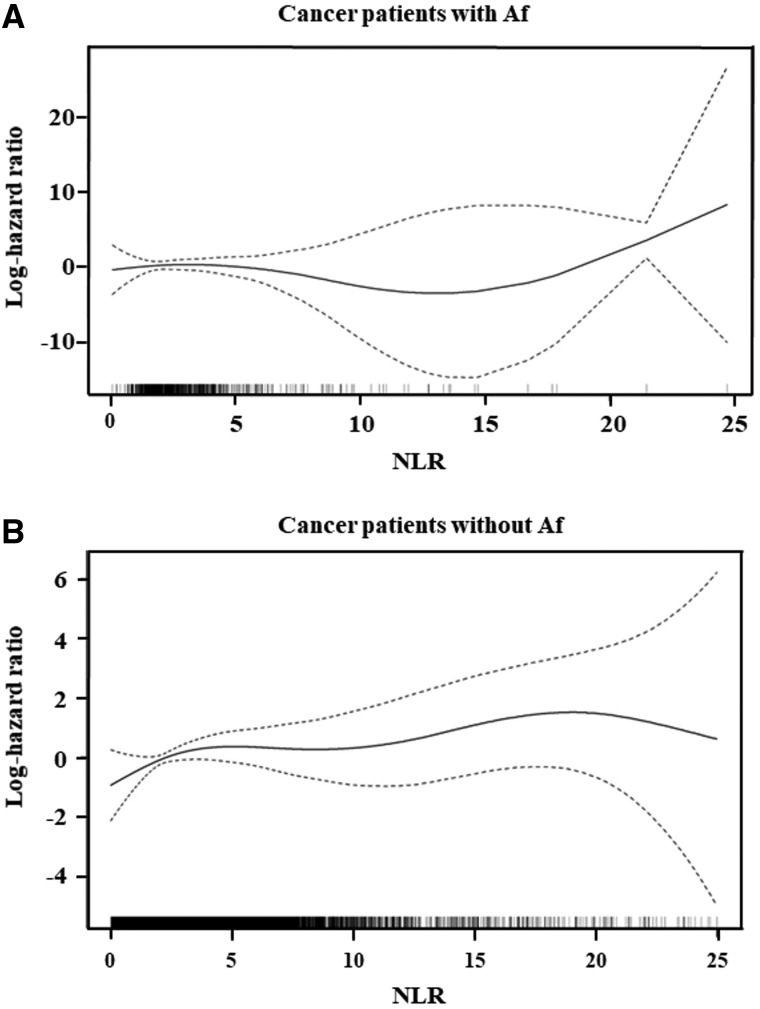
**Smoothing spline analysis for NLR**. Change of the log-hazard ratio over the NLR, as estimated by the spline-based Cox proportional hazards model, stratified by cancer types, for patients with (**A**) and without (**B**) Af.

To understand the possibly non-linear relationship between NLR and the risk of ischaemic stroke, we conducted a smoothing spline analysis for NLR using a Cox proportional hazards model stratified by cancer type. In Fig. 2, plots of the log-hazard ratios over NLR are presented for the Af (A) and non-Af (B) groups, along with pointwise two-tailed 95% confidence bands. Although there was an apparent higher risk for Af patients with NLR >15 (Fig. 2A), the confidence band for the log-hazard ratio curve was very wide. This indicates that the number of events for Af patients was too small to draw any strong conclusions about this relationship.

We next examined this relationship among patients without Af. The log-hazard ratio curve for NLR increased linearly up to almost 5, and was flat up to around 15 (Fig. 2B). Non-Af patients with NLR >15 appeared to have a substantially higher risk of ischaemic stroke, suggesting that NLR might be useful to predict ischaemic stroke in cancer patients without Af. The profile shown in Fig. 2B suggests that non-Af patients can be classified into three subclasses of prognosis, according to cut-off values of NLR around 5 and 15: the low (NLR < 5), middle (5 ≤ NLR ≤ 15) and high (15 < NLR) NLR groups.

### Cancer patients with elevated NLR had advanced cancer stage, regardless of Af status

The clinical backgrounds of the three NLR groups in Af patients are summarized in Supplementary Table 3. The middle NLR group had a significantly high proportion of advanced cancer stage than the low NLR group. However, there were no differences in other stroke risk factors, including older age, hypertension, dyslipidaemia, diabetes mellitus and smoking. Blood examination revealed that the high NLR group had significantly higher WBC and neutrophil counts and CRP levels than the low NLR group. The middle NLR group had significantly lower Hb levels than the low NLR group. In addition, the high NLR group had significantly lower lymphocyte counts and albumin levels than the low NLR group.

The clinical backgrounds of the three NLR groups in non-Af patients are summarized in Supplementary Table 4. The proportion of male patients and advanced cancer stage were higher in the middle and high NLR groups than in the low NLR group. There were no differences in other stroke risk factors. Blood examination revealed that the high NLR group had significantly higher WBC and neutrophil counts and CRP levels than the low NLR group. Additionally, the high NLR group had significantly lower lymphocyte counts and Hb and albumin levels than the low NLR group.

These results indicate that cancer patients with elevated NLRs had advanced cancer stages, high WBC and neutrophil counts, high CRP levels, and low lymphocyte counts and albumin levels, regardless of whether or not they had Af.

### For all cancer sites, the high NLR group had fewer patients

Next, we examined the number of patients with cancer at each cancer site in the three NLR groups, in both the presence and absence of Af (Supplementary Tables 5 and 6). For all cancer sites, the higher NLR groups had fewer patients.

### NLR predicted ischaemic stroke incidence in cancer patients with and without Af

We re-examined the effects of NLR on ischaemic stroke incidence in cancer patients with and without Af. The spline-based Cox analysis, presented in Fig. 2, was conducted for this purpose. However, Fig. 2 suggests that the spline estimate for cancer patients with Af might be unreliable because of the small number of stroke events. To investigate the association between NLR and ischaemic stroke in cancer patients with and without Af, we applied more stable statistical models using the three subclasses of NLR that were suggested by the spline analysis. Table 3 presents the results of multivariate Cox analyses, stratified separately by cancer site, for cancer patients with and without Af. For cancer patients without Af, the high NLR group had a significantly higher risk of ischaemic stroke than the low NLR group. Although the risk of the middle NLR group was not significantly higher than the low NLR group, the risk tended to monotonically increase as NLR increased in cancer patients without Af. For cancer patients with Af, neither the high nor middle NLR group was significantly different from the low NLR group. However, the estimates of all regression coefficients were consistent with those of patients without Af. Thus, the non-significant results in patients with Af might be caused by the small number of stroke events, suggesting no differences in the prognostic capacity of NLR between Af and non-Af patients. Then, we also conducted the Cox regression analysis by pooling patients with and without Af, which led to consistent results. That is, consistently with our finding by the survival tree analysis (Fig. 1) and Table 3, Af and high NLR were significant risk factor for cancer-associated stroke (Supplementary Table 7). The results of the Cox regression analysis with the propensity-score confounder adjustment are presented in Supplementary Table 8 as a sensitivity analysis of the confounder-adjusted analysis given in Table 3 for non-Af patients. The generalized propensity scores for the middle and high NLR groups were estimated by the proportional odds logistic regression model for ordinal responses and the generalized propensity scores were adjusted by including them in the Cox regression as explanatory variables. The significant association of NLR was observed with the propensity-score adjustment and it strengthened the results given in Table 3 for non-Af subjects.

**Table 3:** Stage-stratified Cox regression analyses of NLR in cancer patients with and without Af

	Af group (*n *=* *753)		Non-Af group (*n *=* *17 464)	
	HR (95% CI)	*P*-value	HR (95% CI)	*P*-value
Univariate analysis				
Middle NLR group (5 ≤ NLR ≤ 15)	0.643 (0.081–5.101)	0.676	1.426 (0.659–3.083)	0.367
High NLR group (NLR > 15)	4.025 (0.475–34.080)	0.201	6.240 (1.877–20.741)	0.003
Multivariate analysis				
Middle NLR group (5 ≤ NLR ≤ 15)	0.709 (0.087–5.768)	0.747	1.503 (0.695–3.250)	0.301
High NLR group (NLR > 15)	11.598 (0.953–141.181)	0.055	7.877 (2.351–26.389)	0.001
Age, years, per 1 increase	1.020 (0.965–1.077)	0.491	1.048 (1.019–1.078)	0.001
Hypertension	1.414 (0.399–5.018)	0.592	1.856 (1.002–4.437)	0.0049
Dyslipidaemia	2.142 (0.638–7.194)	0.218	1.294 (0.656–2.552)	0.456
Diabetes mellitus	1.162 (0.374609)	0.795	1.696 (0.915–3.144)	0.093
Drinking history	0.608 (0.129–2.864)	0.530	0.956 (0.373–2.451)	0.925

Af, atrial fibrillation; CI, confidence interval; HR, hazard ratio; NLR, neutrophil-to-lymphocyte ratio.

### High NLR was associated with ischaemic stroke regardless of cancer stage

We finally examined the association between cancer stage and NLR. In Supplementary Fig. 4, the geometric means are shown, along with their confidence intervals by cancer site and cancer stage (advanced, stage III or IV; non-advanced, stage 0–II). Supplementary Fig. 4 indicates that NLRs depended on cancer sites, and that patients with advanced stages consistently had higher NLRs than patients with non-advanced stages, over all cancer sites. The two-way ANOVA revealed a statistically significant association between cancer stage and NLR (*P* < 0.0001). These findings are consistent with previous studies.^21^ However, cancer stage-stratified Cox regression analyses (Table 3) revealed that high NLR was associated with ischaemic stroke, indicating that patients with high NLR at cancer diagnosis had a higher risk of ischaemic stroke, regardless of cancer stage.

### Impact of administration to antibiotics, radiotherapy or chemotherapy on findings

Among 18 217 subjects analysed, 693 subjects were administrated by antibiotics, radiotherapy or chemotherapy at baseline. It might affect the NLR value and then might have some impacts on our findings. To address this concern, we conducted subgroup analyses of 17 524 patients excluding the 693 administrated subjects in our key analyses. In Supplementary Table 9, we compared baseline characteristics between the sub-population and the excluded patients. There were no substantial differences between them observed, suggesting no systematic discrepancy between the original population and the sub-population. In Supplementary Fig. 5, we present the log-hazard ratio of NLR over time estimated by the spline-based Cox regression applied to the sub-population. A consistent picture was obtained to the analysis for the original population in Fig. 2A and B. In Supplementary Table 10, the Cox regression analysis applied to the sub-population was presented, which was a counterpart of Table 3. The statistical significant association of high NLR was preserved in the analyses for the sub-population for non-Af patients and the administration of these drugs had very little influence on the main findings of us.

## Discussion

In a large, heterogeneous cancer registry, we evaluated the predictive capacity of NLR at cancer diagnosis for ischaemic stroke within 2 years of diagnosis. Elevated NLR at diagnosis was associated with a higher incidence of ischaemic stroke among cancer patients, regardless of cancer site and stage. NLR at cancer diagnosis might thus be useful for identifying patients at high risk of ischaemic stroke, allowing us to apply preventive medicine and reduce morbidity.

A few studies have reported associations between NLR and cancer-associated stroke incidence. Chen et al.^22^ reported that elevated D-dimer, total prostate-specific antigen and NLR were independent risk factors of ischaemic stroke among patients with prostate cancer, though this study did not adjust for cancer stage. In contrast, we used data on cancer stage, cancer site, and duration from diagnosis to ischaemic stroke, and applied survival tree analysis and multivariate Cox hazard regression models. We also adjusted for confounding factors, including cancer site and stage, to minimize the risk of confounding bias in the large, heterogeneous study population.

Increased NLR has been associated with poor prognosis in various kinds of cancer, with a cut-off value of 2–5.^23^ In the present study, NLR ≥ 5 was considered to be ‘increased’. Increased NLR in cancer patients is considered to result from systemic inflammatory responses evoked by tumour cells. Increased numbers of neutrophils contribute to tumour expansion because neutrophils produce cytokines, which enhance angiogenesis, and many kinds of ligands, which promote tumour progression and metastasis.^24^ However, under chronic inflammation, activated neutrophils generate extracellular net-like structures (so-called neutrophil extracellular traps). These structures consist of intracellular components, such as decondensed chromatin and histones, and contribute to thrombus formation.^25^ Moreover, inflammatory responses increase the numbers of neutrophil-derived microparticles, which also contribute to thrombosis.^26^ Activated neutrophils might therefore be involved in thrombogenesis in cancer patients with elevated NLRs. In the present study, increased NLR was correlated with elevated CRP and D-dimer levels among cancer patients (Supplementary Fig. 2). These data support that that elevated NLR may be associated with not only inflammatory responses but also thrombus formation/degradation.

In the current study, patients with high NLRs (>15) had significantly more advanced cancer stages, and higher WBC and neutrophil counts and CRP levels, compared with the low NLR group (Supplementary Tables 3 and 4). Furthermore, the high NLR group had significantly lower lymphocyte counts and Hb and albumin levels. In addition, NLR had a significant negative correlation with BMI (Supplementary Fig. 3). Patients with advanced cancer showing these blood biomarkers are thought to represent undernutrition.^27^ Advanced cancers often cause detrimental changes to metabolism and body composition via chronic inflammation; this phenomenon is called cachexia.^28^ High NLRs in cancer patients might therefore reflect chronic inflammation and cachexic status.

Our findings raise the question: Should cancer patients with elevated NLRs be given preventive care? At present, there are limited guidelines for managing cancer-associated stroke. In the present study, the median time from cancer diagnosis to ischaemic stroke was 187 days. This result was consistent with a report by Navi et al.,^29^ which indicated that patients newly diagnosed with cancer had increased ischaemic stroke risk (especially during the first 6 months). Together, these results suggest that physicians should pay attention to ischaemic stroke incidence in newly diagnosed cancer patients.

In our heterogeneous study population, cancer patients with Af had a higher risk of ischaemic stroke than patients without Af (Fig. 1A and B). Based on clinical guidelines from the American College of Cardiology/American Heart Association, patients with Af and elevated CHA_2_DS_2_-VASc scores are recommended to take oral anticoagulants.^30^ When patients are eligible for anticoagulants and have low risk of bleeding, physicians should therefore consider anticoagulant administration.^31^

### Limitations

First, this study used the diagnostic codes in electronic medical records, which may be inaccurate. Second, this study was conducted in a single university hospital and analysed retrospectively. Third, the number of events in each cancer type was relatively low (Table 1), and it was thus impossible to address whether the effects of NLR depend on cancer types. Fourth, we could not show any significant differences on Af population due to the small number of patients with Af. Fifth, we could not assess how many patients had antithrombotic treatment when they were enrolled in the Osaka University cancer registry or whether Af patients received appropriate anticoagulant drugs at the onset of stroke. Finally, our study was unable to distinguish the effects of cancer treatment (radiation therapy, chemotherapy, and surgery) from the risk of stroke. Further multicentre, prospective, longitudinal studies are therefore needed to elucidate the predictive capacity of the NLR. We, therefore, had started a multicentre prospective observational study, in which acute stroke patients with cancer were enrolled. Ischaemic Stroke in Patients with Cancer and Neoplasia study (SCAN study; UMIN ID: UMIN000043473) involves Osaka University Hospital and seven other hospitals in Japan. We are investigating new methods of diagnosis and treatment for cancer-associated stroke.

## Conclusions

Higher NLR at cancer diagnosis was associated with a greater incidence of ischaemic stroke among cancer patients. NLR is easy and cheap to measure. NLR at diagnosis might be useful for identifying patients at high risk for ischaemic stroke, thus allowing the use of preventive treatments, which might reduce ischaemic stroke incidence and mortality.

## Supplementary material

Supplementary material is available at *Brain Communications* online.

## Supplementary Material

fcab071_Supplementary_Data

## Abbreviations

Af =: atrial fibrillation
BMI =: body mass index
CRP =: C-reactive protein
Hb =: haemoglobin
NLR =: neutrophil-to-lymphocyte ratio
WBC =: white blood cell
