# Supplementary material for: High neutrophil/lymphocyte ratio at cancer diagnosis predicts incidence of stroke in cancer patients
Source: Brain Commun. 2021 Apr 9;3(2):fcab071. doi: 10.1093/braincomms/fcab071 (PMC8062330; doi:10.1093/braincomms/fcab071)

**Supplementary Online Content**

Tomohiro Kawano, Tsutomu Sasaki, Yasufumi Gon, Takaya Kitano, Hideaki Kanki, Kenichi Todo, Munehisa Shimamura, Yasushi Matsumura, Ao Huang, Hattori, Hideki Mochizuki. High Neutrophil-to-Lymphocyte Ratio at Cancer Diagnosis Predicts the Incidence of Ischemic Stroke Among Cancer Patients

**Supplementary Table 1. Ischemic stroke incidence for patients with cancer at each site**

**Supplementary Table 2. The number and frequency of ischemic stroke patients with cancer at each site**

**Supplementary Table 3. Clinical characteristics of cancer patients with Af at three different levels of NLR**

**Supplementary Table 4. Clinical characteristics of cancer patients without Af at three different levels of NLR**

**Supplementary Table 5. Number of patients with cancer at each site in patients with Af**

**Supplementary Table 6. Number of patients with cancer at each site in patients without Af**

**Supplementary Table 7. The stage-stratified Cox regression analyses of NLR in pooling patients with Af and without Af**

**Supplementary Table 8. The Cox regression analysis with the propensity score adjustment for patients without Af**

**Supplementary Table 9. Comparison of clinical characteristics between patients with and without antibiotics, radiotherapy or chemotherapy administrated**

**Supplementary Table 10. Stage-stratified Cox regression analyses of NLR in subpopulation with and without A**f

**Supplementary Figure 1. Temporal distribution of ischemic stroke (A) and frequency of ischemic stroke adjusted by the number of cancer survivors (B)**

**Supplementary Figure 2. Correlation of NLR with CRP (A) and D-dimer (B) levels among cancer patients.**

**Supplementary Figure 3. The association of NLR with BMI among cancer patients**

**Supplementary Figure 4. The NLR levels of patients in the advanced (stage III or IV, upper rows) and non-advanced (stage 0­–II, lower rows) states in each cancer type.**

**Supplementary Figure 5. Change of the log-hazard ratio over the NLR, as estimated by the spline-based Cox proportional hazards model, stratified by cancer types, for subpopulation with Af (A) and without Af (B)**.

This supplementary material has been provided by the authors to give readers additional information about their work.

**Supplementary Table 1. Ischemic stroke incidence for patients with cancer at each site**

| Cancer site | Ischemic stroke incidence in the Af group  (*n*=753) | Ischemic stroke incidence in the non-Af group  (*n*=17,464) | Total  (*n*=18,217) |
| --- | --- | --- | --- |
| Breast | 0/30 | 2/2291 | 2/2321 |
| Uterine | 0/24 | 2/1922 | 2/1946 |
| Gastric | 0/76 | 4/1577 | 4/1653 |
| Colorectal | 5/73 | 1/1377 | 6/1450 |
| Prostate | 1/62 | 4/1371 | 5/1433 |
| Lung | 2/105 | 10/1178 | 12/1283 |
| Esophageal | 1/88 | 8/1083 | 9/1171 |
| Oropharyngeal | 2/33 | 6/825 | 8/858 |
| Hepatic | 0/50 | 1/719 | 1/769 |
| Pancreatic | 1/22 | 5/579 | 6/601 |
| Others* | 4/190 | 10/4542 | 14/4732 |

Af, atrial fibrillation. *Includes skin cancer, renal cancer, thyroid cancer, ovarian cancer, bladder cancer, malignant lymphoma, biliary tract cancer, laryngeal cancer, soft connective tissue cancer, spinal cord tumors, cancer of unknown primary origin, eye cancer, peritoneal cancer, bone cancer, small intestinal cancer, maxillary sinus cancer, testicular cancer, mediastinal cancer, ureter cancer, nasal cancer, vulvar cancer, parathyroid cancer, vaginal cancer, penile cancer, scrotal cancer, pineal tumor, anus cancer, tracheal cancer, peripheral nerve tumor, and choriocarcinoma.

**Supplementary Table 2. The number and frequency of ischemic stroke patients with cancer at each site**

| Cancer site | Number (%)  (n=69) |
| --- | --- |
| Breast | 2 (3) |
| Uterine | 2 (3) |
| Gastric | 4 (6) |
| Colorectal | 6 (9) |
| Prostate | 5 (7) |
| Lung | 12 (17) |
| Esophageal | 9 (13) |
| Oropharyngeal | 8 (12) |
| Hepatic | 1(1) |
| Pancreatic | 6 (9) |
| Others* | 14 (20) |

*Includes skin cancer, renal cancer, thyroid cancer, ovarian cancer, bladder cancer, malignant lymphoma, biliary tract cancer, laryngeal cancer, soft connective tissue cancer, spinal cord tumors, cancer of unknown primary origin, eye cancer, peritoneal cancer, bone cancer, small intestinal cancer, maxillary sinus cancer, testicular cancer, mediastinal cancer, ureter cancer, nasal cancer, vulvar cancer, parathyroid cancer, vaginal cancer, penile cancer, scrotal cancer, pineal tumor, anus cancer, tracheal cancer, peripheral nerve tumor, and choriocarcinoma.

**Supplementary Table 3. Clinical characteristics of cancer patients with Af at three different levels of NLR**

| Variable | **Low group**  **NLR<5**  **(*n*=661)** | **Middle group**  **5≤NLR≤15**  **(*n*=85)** | **High group**  **15<NLR**  **(*n*=7)** | *P-*value |
| --- | --- | --- | --- | --- |
| Age, years | 72 [66–78] | 71 [66–79] | 71 [62–75] | 0.4182 |
| Male (%) | 486 (74) | 52 (61) | 5 (71) | 0.0575 |
| Stage of cancer |  |  |  |  |
| Nonadvanced (%) | 455 (71) | 39 (50) | 6 (86) | <.001 |
| Advanced (%) | 182 (29) | 39 (50) | 1 (14) |  |
| Hypertension (%) | 371 (56) | 41 (48) | 4 (57) | 0.3853 |
| Dyslipidemia (%) | 216 (33) | 22 (26) | 1 (14) | 0.2467 |
| Diabetes mellitus (%) | 274 (41) | 38 (45) | 3 (43) | 0.8483 |
| Smoking (%) | 199 (38) | 18 (30) | 3 (60) | 0.2962. |
| WBCs,  ×10^3^/µL | 4.9 [4.9–7.4] | 8.3 [6.4–10.8] | 13.8 [12.2–20.7] | <.0001 |
| Neutrophils,  ×10^3^/µL | 3.6 [2.9–4.7] | 6.4 [5.2–8.5] | 12.5 [10.8–17.8] | <.0001 |
| Lymphocytes, ×10^3^/µL | 1.6 [1.3–2.0] | 1.0 [0.7–1.2] | 0.60 [0.47–0.68] | <.001 |
| Hb, g/dL | 13.4 [12.0–14.5] | 11.6 [10.1–13.3] | 12.8 [9.5–14.9] | <.0001 |
| CRP, mg/L | 0.20 [0.08–0.67] | 0.55 [0.16–3.02] | 4.05 [0.25–9.96] | <.0001 |
| Alb, mg/L | 3.9 [3.6–4.2] | 3.6 [3.2–3.9] | 3.2 [2.8–3.4] | <.05 |

Data are given as median [interquartile range] or number (percentage). NLR, neutrophil-to-lymphocyte ratio; n.s., not significant; WBC, white blood cell; Hb, hemoglobin; CRP, C-reactive protein; Alb, albumin.

**Supplementary Table 4. Clinical characteristics of cancer patients without Af at three different levels of NLR**

| Variable | **Low group**  **NLR<5**  **(*n*=15,397)** | **Middle group**  **5≤NLR≤15**  **(*n*=1891)** | **High group**  **15<NLR**  **(*n*=176)** | *P-*value |
| --- | --- | --- | --- | --- |
| Age | 65 [54–73] | 65 [53–74] | 64 [49–73] | 0.2033 |
| Male (%) | 7671 (50) | 1038 (55) | 96 (55) | <.001 |
| Stage of cancer |  |  |  |  |
| Nonadvanced (%) | 10681 (72) | 808 (46) | 58 (38) | <.0001 |
| Advanced (%) | 4081 (28) | 960 (54) | 95 (62) |  |
| Hypertension (%) | 3462 (22) | 452 (24) | 39 (22) | 0.3670 |
| Dyslipidemia (%) | 2242 (15) | 249 (13) | 26 (15) | 0.2631 |
| Diabetes mellitus (%) | 3003 (20) | 380 (20) | 39 (22) | 0.5723 |
| Smoking (%) | 3372 (32) | 380 (32) | 39 (35) | 0.7613 |
| WBCs,  ×10^3^/µL | 6.0 [4.9–7.3] | 8.4 [6.6–10.9] | 12.4 [8.7–20.2] | <.0001 |
| Neutrophils,  ×10^3^/µL | 3.7 [2.8–4.7] | 6.7 [5.2–8.8] | 11.3 [8.0–18.2] | <.0001 |
| Lymphocytes, ×10^3^/µL | 1.7 [1.3–2.1] | 1.0 [0.76–1.26] | 0.57 [0.40–0.83] | <.05 |
| Hb, g/dL | 13.3 [12.1–14.3] | 12.3 [10.8–13.7] | 11.5 [10–13] | <.0001 |
| CRP, mg/L | 0.14 [0.06–0.54] | 1.12 [0.2–4.1] | 3.58 [0.44–9.31] | <.0001 |

Data are given as median [interquartile range] or number (percentage). NLR, neutrophil-to-lymphocyte ratio; n.s., not significant; WBC, white blood cells; Hb, hemoglobin; CRP, C-reactive protein.

**Supplementary Table 5. Number of patients with cancer at each site in patients with Af**

| Cancer site | **Low group**  **NLR<5**  **(*n*=661)** | **Middle group**  **5≤NLR≤15**  **(*n*=85)** | **High group**  **15<NLR**  **(*n*=7)** |
| --- | --- | --- | --- |
| Breast | 30 | 0 | 0 |
| Colorectal | 60 | 12 | 1 |
| Esophageal | 74 | 13 | 1 |
| Gastric | 68 | 7 | 1 |
| Hepatic | 47 | 3 | 0 |
| Lung | 96 | 9 | 0 |
| Oropharyngeal | 29 | 3 | 1 |
| Pancreas | 21 | 0 | 1 |
| Prostate | 59 | 3 | 0 |
| Uterus | 19 | 5 | 0 |
| Others | 29 | 3 | 1 |

NLR, neutrophil-to-lymphocyte ratio.

**Supplementary Table 6. Number of patients with cancer at each site in patients without Af**

| Cancer site | **Low group**  **NLR<5**  **(*n*=15,397)** | **Middle group**  **5≤NLR≤15**  **(*n*=1891)** | **High group**  **15<NLR**  **(*n*=176)** |
| --- | --- | --- | --- |
| Breast | 2136 | 142 | 13 |
| Colorectal | 1230 | 140 | 7 |
| Esophageal | 940 | 136 | 7 |
| Gastric | 1426 | 142 | 9 |
| Hepatic | 646 | 73 | 0 |
| Lung | 972 | 186 | 20 |
| Oropharyngeal | 726 | 88 | 11 |
| Pancreas | 471 | 99 | 9 |
| Prostate | 1305 | 63 | 3 |
| Uterus | 1748 | 168 | 6 |
| Others | 3797 | 654 | 91 |

NLR, neutrophil-to-lymphocyte ratio.

**Supplementary Table 7. The stage-stratified Cox regression analyses of NLR in pooling patients with Af and without Af**

|  | HR (95% CI) | *P*-value |
| --- | --- | --- |
| Univariate analysis | | |
| Middle NLR group (5≤NLR≤15) | 1.260 (0.615–2.580) | .527 |
| High NLR group (NLR>15) | 6.573 (2.333–18.521) | <.001 |
| Multivariate analysis | | |
| Middle NLR group (5≤NLR≤15) | 1.358 (0.664–2.779) | .402 |
| High NLR group (NLR>15) | 9.459 (3.325–26.914) | <.001 |
| Atrial fibrillation | 3.782 (2.096-6.825) | <.001 |
| Age, years, per 1 increase | 1.040 (1.015–1.067) | .002 |
| Hypertension | 1.677 (0.966–2.913) | .066 |
| Dyslipidemia | 1.577 (0.891-2.791) | 0.118 |
| Diabetes mellitus | 1.592 (0.932-2.718) | 0.089 |
| Drinking history | 0.926 (0.417-2.057) | 0.850 |

**Supplementary Table 8. The Cox regression analysis with the propensity score**

|  | HR (95% CI) | *P*-value |
| --- | --- | --- |
| Middle NLR group (5≤NLR≤15) | 1.508 (0.687–3.306) | .306 |
| High NLR group (NLR>15) | 8.551 (2.547–28.712) | .001 |

Age, hypertension, dyslipidemia, diabetes mellitus, drinking history, and cancer stage were used to estimate the propensity score.

**Supplementary Table 9. Comparison of clinical characteristics between patients with and without antibiotics, radiotherapy or chemotherapy administrated**

|  | Excluded patients  (*n*=693) | Subpopulation  (*n*=17,524) |
| --- | --- | --- |
| Age, years | 69 [61–76] | 65 [55–73] |
| Male (%) | 428 (62) | 8920 (51) |
| Stage of cancer |  |  |
| Nonadvanced (%)  (0, Ⅰ, and Ⅱ) | 342 (51) | 11706 (69) |
| Advanced (%)  (Ⅲ and Ⅳ) | 323 (49) | 5035 (30) |
| Hypertension (%) | 166 (24) | 4203 (24) |
| Dyslipidemia (%) | 110 (16) | 2646 (15) |
| Diabetes mellitus (%) | 158 (23) | 3579 (20) |
| Smoking (%) | 146 (33) | 3865 (33) |
| Drinking history (%) | 36 (5) | 1409 (8) |
| Af (%) | 31 (4) | 722 (4) |
| BMI | 21.8 [19.6-24.0] | 22.2 [20.0-24.5] |
| WBCs,  ×10^3^/µL | 6.3 [5.0–8.3] | 6.2 [5.0–7.7] |
| Neutrophils,  ×10^3^/µL | 4.2 [3.0–6.0] | 3.9 [2.9–5.1] |
| Lymphocytes, ×10^3^/µL | 1.4 [1.0–1.8] | 1.6 [1.2–2.0] |
| NLR | 3.0 [2.0–5.1] | 2.4 [1.7–3.5] |

Data are given as median [interquartile range] or number (percentage). Af, atrial fibrillation; BMI, body mass index, WBC, white blood cell, NLR, neutrophil-to-lymphocyte ratio.

**Supplementary Table 10. Stage-stratified Cox regression analyses of NLR in subpopulation with and without A**f

|  | Af group | | Non-Af group | |
| --- | --- | --- | --- | --- |
|  | HR (95% CI) | *P*-value | HR (95% CI) | *P*-value |
| Univariate analysis | | | | |
| Middle NLR group (5≤NLR≤15) | 0.867 (0.106–7.10) | .894 | 0.849 (0.300–2.401) | .758 |
| High NLR group (NLR>15) | 8.580 (1.030–71.60) | .047 | 5.402 (1.267–23.026) | .023 |
| Multivariate analysis | | | | |
| Middle NLR group (5≤NLR≤15) | 0.942 (0.110–8.08) | .956 | 0.942 (0.333–2.662) | .910 |
| High NLR group (NLR>15) | 31.200 (2.270–428.00) | .010 | 6.863 (1.599–29.456) | .010 |
| Age, years,  per 1 increase | 0.998 (0.941–1.06) | .935 | 1.052 (1.020–1.085) | .001 |
| Hypertension | 2.630 (0.532–13.00) | .235 | 2.245 (1.151–4.379) | .018 |
| Dyslipidemia | 2.580 (0.665-9.99) | 0.171 | 1.344 (0.648-2784) | .427 |
| Diabetes mellitus | 0.775 (0.228-2.64) | 0.684 | 1.484 (0.758-2.907) | 0.250 |
| Drinking history | 0.560 (0.110-2.84) | 0.484 | 0.842 (0295-2.00) | 0.747 |

**Supplementary Figure 1. Temporal distribution of ischemic stroke (A) and frequency of ischemic stroke adjusted by the number of cancer survivors (B).**

**
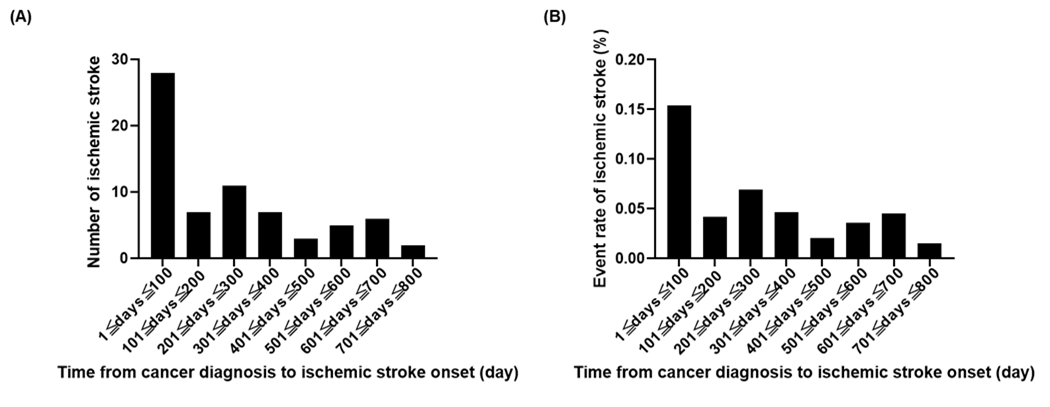
**

NLR, neutrophil-to-lymphocyte ratio

**Supplementary Figure 2. Correlation of NLR with CRP (A) and D-dimer (B) levels among cancer patients.**

**
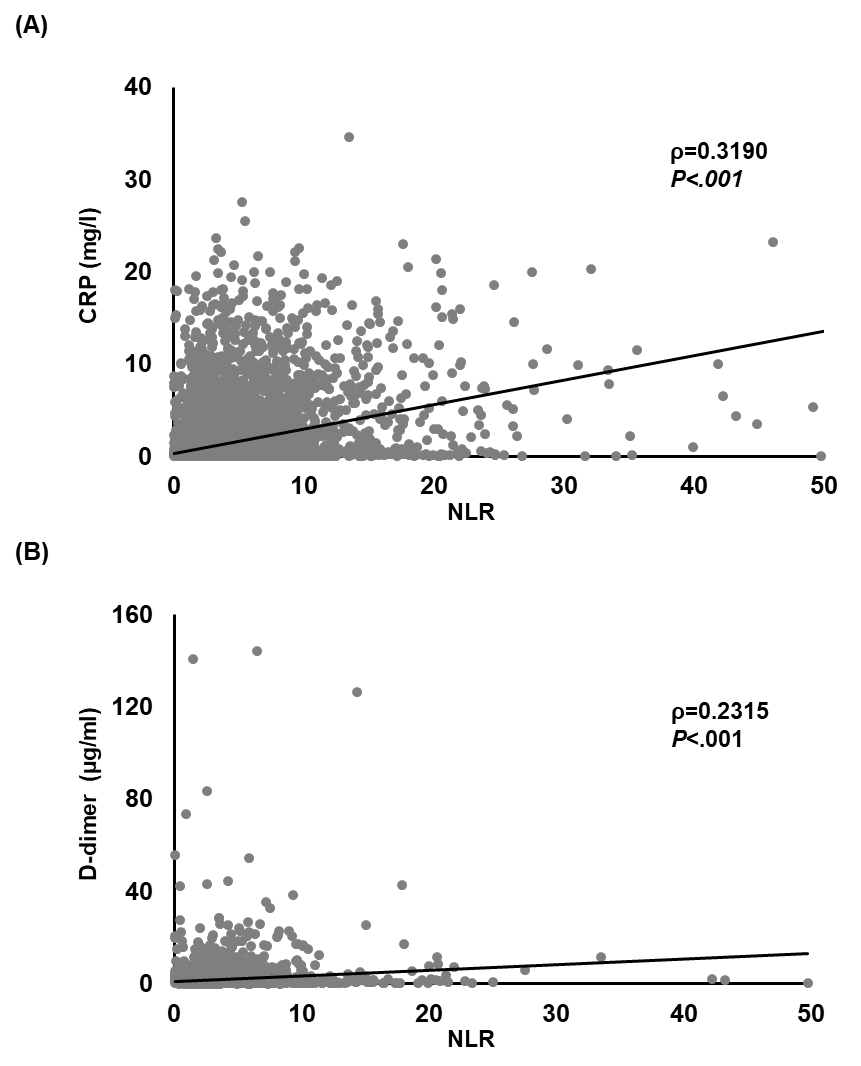
**

CRP, C reactive protein; NLR, neutrophil-to-lymphocyte ratio

**Supplementary Figure 3. The association of NLR with BMI among cancer patients**

**
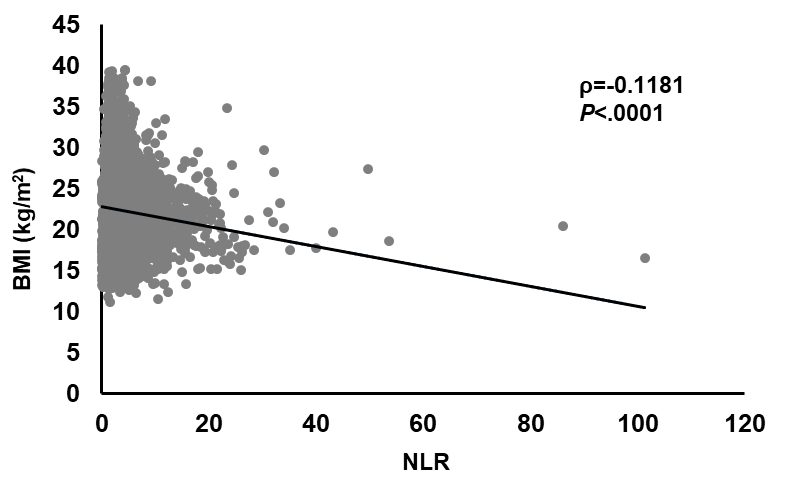
**

NLR, neutrophil-to-lymphocyte ratio; BMI, body mass index

**Supplementary Figure 4. The NLR levels of patients in the advanced (stage III or IV, upper rows) and non-advanced (stage 0­–II, lower rows) states in each cancer type.**


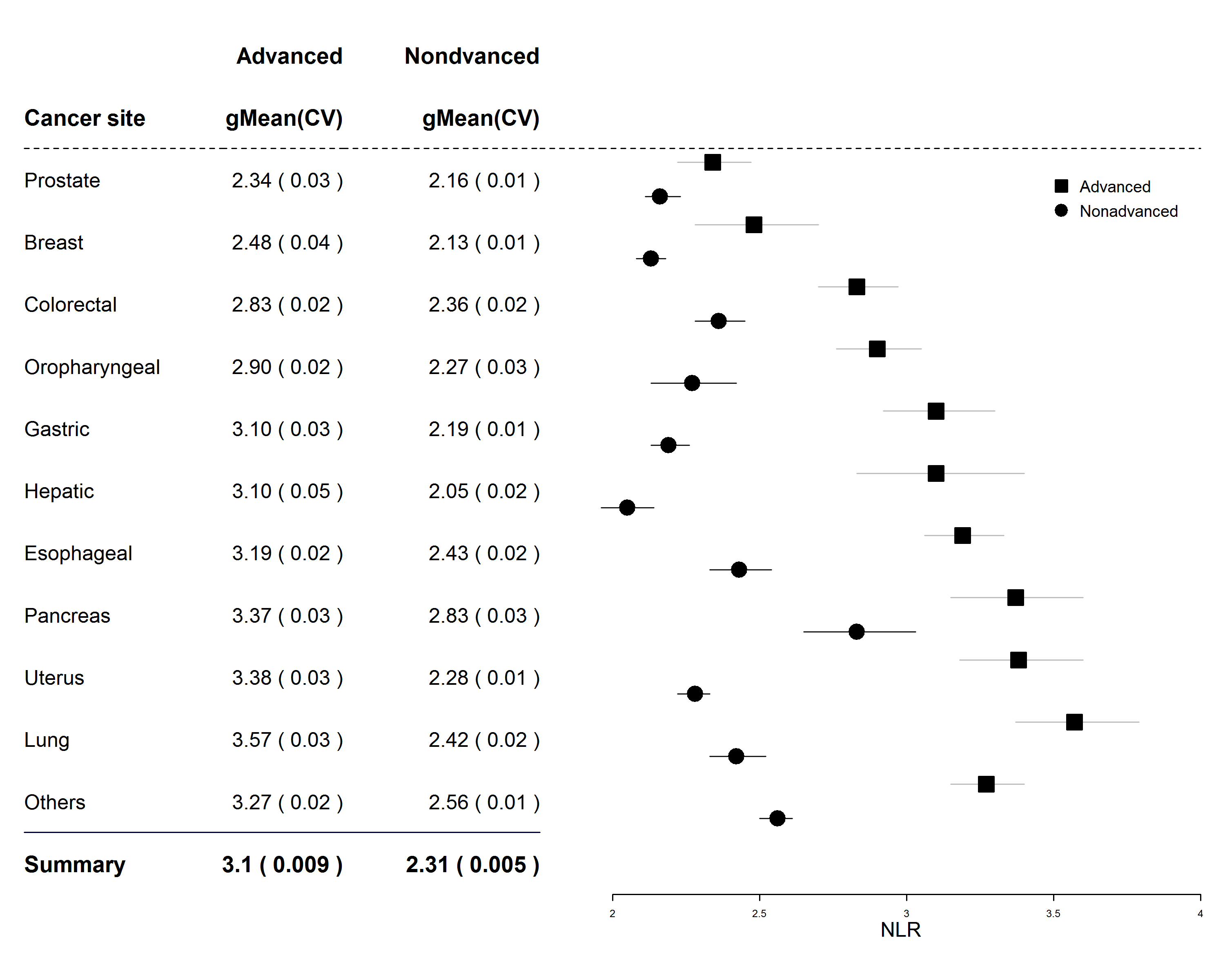


gMEAN, geometric mean; CV, coefficient of variation.

**Supplementary Figure 5. Change of the log-hazard ratio over the NLR, as estimated by the spline-based Cox proportional hazards model, stratified by cancer types, for subpopulation with Af (A) and without Af (B)**.


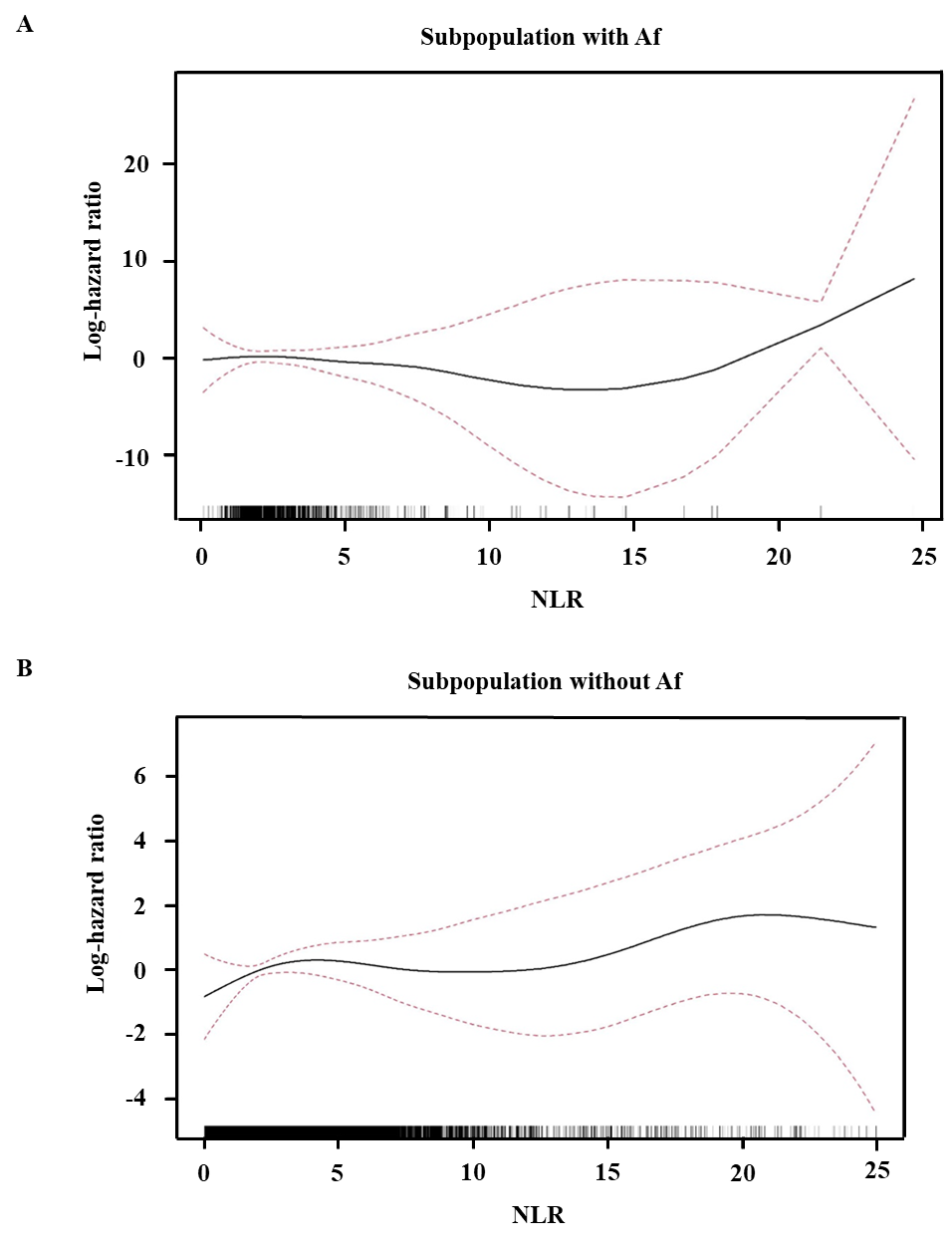

Supplement: fcab071_Supplementary_Data [file fcab071_supplementary_data.docx]
